# The role of pit lake thermal dynamics on the thermal performance of ground heat exchangers

**DOI:** 10.1038/s41598-024-69225-6

**Published:** 2024-08-19

**Authors:** Mauricio Carcamo-Medel, Guillermo Narsilio, Raul Fuentes

**Affiliations:** 1 Department of Infrastructure Engineering, The University of Melbourne, Parkville, 3010 Australia; 2 Institute of Geomechanics and Underground Technology, RWTH Aachen University, 52074 Aachen, Germany

**Keywords:** Shallow geothermal, Mine closure, Mine end uses, Pit lake, Numerical modelling, Ground heat exchangers, Geothermal energy, Civil engineering

## Abstract

The addition of ground heat exchangers (GHEs) to a pit lake’s basin has the potential for abundant, clean and renewable geothermal energy extraction using shallow geothermal systems. Basin-embedded GHEs avoid direct interaction with mine water, which has been shown to impact efficiency and longevity in mine open-loop geothermal systems negatively. The now accelerated closure of open-pit coal mines presents itself as an opportunity to use this technology. However, no guidelines currently exist for designing or operating GHEs embedded in the sediment of water bodies. Furthermore, the two-way coupling between the complex annual thermal fluid dynamics that lakes are naturally subjected to and heat fluxes on the sediments and the GHE system has not been explored. In this study, we develop and validate finite element models to assess the relevance of lake thermal stratification in the performance of a geothermal system embedded in water bodies basins, e.g., on open-pit mine closures, under temperate residential thermal loads. The results show that the pit lake’s role as a thermal sink improves significantly when the lake’s thermal dynamics are accounted for, with an increase of up to 292% in the lake’s available energy budget. A minor variation in energy budget (~8%) was found whether the lake is modelled explicitly or simplified as a transient Dirichlet temperature boundary condition. This small difference vanishes if horizontal circulation along the lake is considered, highlighting the lake’s thermal energy potential. Finally, the impact on the GHE Coefficient of Performance (COP) is evaluated, with a maximum of ~15% difference among all cases.

## Introduction

The closure of a mine is an unavoidable milestone in its life cycle. Abandoned mine assets could represent a significant liability for nearby human populations and wildlife after operation ceases^1^. Over the last decades, considerable efforts have been made to improve mine decommissioning procedures^2^. Current standards highlight the critical aspect of closure plans in mining projects, which must be considered from the conceptual design phase and continuously updated throughout the project’s lifespan^3^. Nevertheless, mine closure policies have focused on safety and environmental hazards, with comparatively, little regard for social and economic transition for communities affected by the closure^4,5^.

Among the mine assets, open-pit excavations are a significant source of risks^6^. Abandoned pits flood naturally if the excavation is below the natural groundwater table due to the halt of dewatering activities and additional inflows from precipitation and runoff^7^. The accumulated water may become toxic due to extreme acidity or the presence of heavy metals and contaminate nearby ecosystems if enabling hydrogeological conditions are met^8^. Moreover, the void itself constitutes a safety risk for humans and wildlife^3^. Additional hazards that affect the integrity of the mine and its surroundings, such as instabilities of the pit walls and land subsidence, may also be present^9–11^. While an open-pit becomes a significant risk if left unmanaged, a well-designed pit decommissioning solution minimises risks and could transform the mining legacy into an asset instead of a liability^12^.

**Figure 1:**
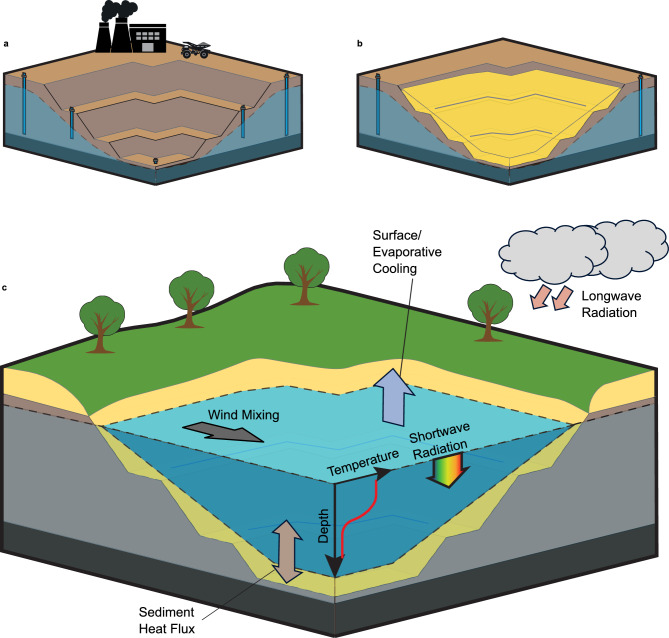
Open-pit mine decommissioning phases into an engineered pit lake. (**a**) End of operation: Pit walls are exposed to the atmosphere and active water pumping maintains the groundwater table below the pit surface. (**b**) Earthmoving works: Pit walls are profiled to guarantee long-term stability; hazardous acidic rocks or self-combusting coal layers are covered using excess overburden or nearby borrowed material. Groundwater table ascent begins once earthworks are completed, and water pumping is gradually phased out. (**c**) End of Rehabilitation: The pit lake is filled, and the land around is rehabilitated to match prospective end-uses. Before land relinquishment, a monitoring phase is carried out to guarantee that the decommissioning targets are consistently met over time. Key physical processes that govern lake dynamics are additionally highlighted in (**c**).

An engineered pit lake is one of the potential solutions for open-pit mine decommissioning, notably for locations where overburden for complete pit backfill is unavailable or prohibitively expensive to achieve^13^. After the mine operation ceases (Fig. 1a), additional earthmoving is typically performed to dump excess overburden material into the pit or to profile pit walls to achieve stability during the void filling and in the long term (Fig. 1b)^14,15^. Unassisted lake filling occurs due to natural processes such as groundwater inflow, precipitation and catchment area runoff, and may take decades^16^. Additional water sources can speed up filling and contribute to pit lake water remediation^16,17^. Finally, following pit lake filling and surrounding land rehabilitation (Fig. 1c), relinquishment of the site should only occur after risk mitigation measures are proven in the long term^3^.

Many challenges remain in the design of pit lakes; one of increasing relevance is developing potential new end uses^6,18^. A technology that shows potential is the addition of a low-enthalpy (shallow) geothermal system in the pit lake for heating or cooling of nearby infrastructure and buildings^18–20^. In a (pit) lake context, a shallow geothermal energy system would extract heat from the lake by direct pumping of lake water (open loop) or by secondary loops laid on the water itself or the lake sediment surface^21,22^. A heat pump (HP) upgrades the fluid temperature and feeds heat to the infrastructure or building through its inner distribution system^21^. The HP cycle may be reversed to provide cooling, where the ground or water body becomes the heat sink.

Large water bodies have a significant budget of low enthalpy geothermal energy^23^. For lakes and water bodies in general, open loop geothermal extraction is a more mature technology compared to close loop, with the former having a more significant number of case studies and design guidelines found in the literature^24,25^. In a mining environment, open-loop geothermal systems have the advantage of more effortless scalability but with the disadvantage of difficulties with plume management, dependence on water availability, and accumulation of chemical or biological precipitates in the heat exchanger, affecting efficiency and operation^22,24,26^. Walls et al. (2021) cite this last disadvantage as one of the leading causes of the cease of operation of geothermal systems installed in underground mines^27^. In contrast, closed-loop systems do not interact directly with fresh or mine water and could extract or reject heat along a larger water volume, or ground volume, if needed. On the other hand, difficulties with capacity scaling and reduced efficiency, due to the additional required temperature gradient between the loop and its surrounding media, are present^25,26^.

A particular case arises for some decommissioned pit lakes, where the closure phase provides an opportunity for extensive ground heat exchanger (GHE) installation during earthmoving works. For natural water bodies, Spitler et al.^25^ categorised heat exchangers buried in their sediments as a bottom sediment heat exchanger (BSHE), able to extract or reject heat both from the sediment as well as the upper water body. A closed loop system embedded in the coast seabed in Botngård, Norway, produces a maximum of 1 MW of heating for a district heating network (DHN) using 20km of high density polyethylene (HDPE) pipes, with a maximum output of ${{50\, W\,m}}^{{-1}}$^25,28^. Similarly, a BSHE system is described in^29^ where 7.8 km of coaxial heat exchangers are embedded in the seabed in Vaasa, Finland. The system feeds approximately 0.35MW of heat to 43 houses connected through a small DHN, reporting heat outputs around ${{40\,W\,m}}^{{-1}}$ to ${{50\,W\,m}}^{{-1}}$ based on previously reported empirical measurements. Finally, a novel capillary box heat exchanger arrangement is presented in^30^, with experimental/pilot studies performed in Shandong province, China. The performance of the system is reported in terms of its coefficient of performance (COP) and energy efficiency ratio (EER) equal to 2.96/5.39, respectively, which would outperform a typical air source heat pump (ASHP) in the same location. No records of the application of BSHEs in pit lakes have been found in the literature.

While sharing many similarities with the previously presented applications of BSHE, GHE embedded in a pit lake basin could be installed at a much broader range of depths, as open pits can extend hundreds of metres below the surface, with complete access before filling begins. Consequently, the GHE could interact with turbulent shallow waters close to the surface and deeper waters, where mixing capabilities could be limited along the season. Moreover, extensive temporal and spatial scales surround pit mine decommissioning, providing further complexity to the assessment of GHE performance. Under these conditions, numerical modelling allows for the evaluation of the behaviour considering both the relevant physical process, and representative temporal and spatial scales, contributing to the further development of the technology. From the previously presented cases, only^30,31^ has considered 3D numerical modelling for BSHE, using ANSYS-CFD and ANSYS-FLUENT, performing a 3D model of the embedded GHE in the seabed, while simplifying the role of seawater above as a simple Dirichlet temperature boundary condition^31^ or as a Robin convective boundary condition^30^. Whether these boundary conditions are appropriate for modelling BSHE embedded in lakes is not yet understood. Further study of the role of boundary conditions is needed for better estimation of the potential of BSHE in open-pit mine decommissioning.

In this paper, we present the first numerical approach to the interaction between embedded GHE and a pit lake, while focusing on the system efficiency, available thermal budget and carrier fluid temperature output. In particular, we focus on understanding and quantifying the role of boundary conditions, considering the stratified water column commonly found in deep lakes and pit lakes. For this purpose, three two-dimensional (2D) numerical models of varying complexity are developed using the finite element method (FEM) package COMSOL Multiphysics^32^, where GHE embedded at a broad range of depths extract or inject thermal energy at the basin of the pit lake. The model’s capability to simulate lake thermal stratification and thermal exchange between the ground and heat exchangers is verified using well-established software and published data in the literature.

## Methods

### Finite element model and governing equations

A transient finite element approach to model GHEs, developed at the University of Melbourne^33^ and implemented in the FEM package COMSOL Multiphysics^32^, is extended and used to conduct this study. The model was initially developed for 3D assessment of borehole heat exchangers and thermally active piles but has been later expanded to assess horizontal GHE under varied boundary conditions^34^. In principle, the model solves coupled heat transfer and fluid flow equations to model the GHE carrier fluid’s convective heat transfer and its thermal interaction with the HDPE pipe and surrounding ground through conduction. Given the broad range of scales of interest for the thermal transfer model, GHE pipes of a few centimetres in diameter to a pit lake of hundreds of meters of width, this research considers a 2D finite element model to minimise the required computational resources. For this purpose, 2D linear elements are used to solve the heat transfer equation in the ground domain:

1 $$\begin{aligned} \frac{\partial T _{\rm g}}{\partial t} - \nabla \cdot ( \alpha _{\rm{g}} \nabla T _{\rm g}) = 0 \end{aligned}$$

where ${T}_{g}$ is the ground temperature and ${\alpha }_{g}$ is the ground thermal diffusivity, defined as:

2 $$\begin{aligned} \alpha _{\rm g} = \frac{\lambda _{\rm g}}{\rho _{\rm g} C_{p,{\rm g}}} \end{aligned}$$

where ${\rho }_{\rm{g}}$, ${C}_{pg}$ and ${\lambda }_{g}$ are the ground bulk density, specific heat capacity and thermal conductivity, respectively.

This work includes three cases of study, detailed in the next section: Case 1, where only material conduction heat transfer through the lake is considered; Case 2, time-varying lake temperature considered as a boundary condition for the ground, and; Case 3, which explicitly accounts for the turbulent thermal modelling of the lake, including incident shortwave radiation. When the lake thermal dynamics are explicitly included in the model (Case 3), the heat transfer equations must be extended to account for the turbulent thermal diffusivity and solar radiation. The adopted approach follows established geophysical lake and ocean dynamics models for Cartesian coordinates, that is^35,36^:

3 $$\begin{aligned} \frac{\partial T _{\rm w}}{\partial t} - \frac{\partial }{\partial z} \left( \alpha _{\rm w,v}' \frac{\partial T _{\rm w}}{\partial z} \right) - \frac{\partial }{\partial x} \left( \alpha _{\rm w,h}' \frac{\partial T _{\rm w}}{\partial x} \right) = \frac{1}{C_{p,{\rm w}} \rho _{\rm w,0}} \frac{\partial I(z)}{\partial z} \end{aligned}$$

where ${T}_{w}$ corresponds to the lake water temperature, $\rho _{\rm{w,0}}$ is the water density for the reference temperature condition, $C_{pw}$ is the water specific heat capacity, and *I*(*z*) correspond to the incoming shortwave radiation. The effective thermal diffusivity in each direction is defined as:

4 $$\begin{aligned} \alpha _{\rm w,i}' = \alpha _ {\rm {mat-w}} + \alpha _{\rm {turb-i}} \qquad i \in \{{\rm h,v}\} \end{aligned}$$

where the effective thermal diffusivity is calculated considering a material (molecular) thermal diffusivity for the lake $\alpha _{\rm{mat-w}}$ plus a directional turbulent thermal diffusivity $\alpha _{\rm{turb-h}}$ or $\alpha _{\rm{turb-v}}$ for the horizontal and vertical direction, respectively. $\alpha _{{\rm mat}-{{\rm w}}}$ is calculated as:

5 $$\begin{aligned} \alpha _{\rm mat-w} = \frac{\lambda _{\rm w}}{\rho _{\rm w} C_{p,{\rm w}}} \end{aligned}$$

where $\lambda _{w}$ is the water material thermal conductivity. In geophysical modelling for water bodies, given the preponderance of vertical thermal stratification, the vertical thermal diffusivity is calculated as part of the solution of a coupled 1D vertical k-$\varepsilon$ turbulent flow - thermal model, herein modelled using one-way coupling with the 1D finite difference model, SIMSTRAT^37^, as detailed later. On the other hand, horizontal turbulent fluxes processes are resolved on smaller scales than those often attainable in geophysical model grids of water bodies^38^. A common approximation is to consider a horizontal turbulent thermal diffusivity $\alpha _{\rm{turb-h}}$ parameter with a constant value, determined diagnostically from calibration with existing data, with ${10}{{\rm m}}^{2}/{{\rm s}}$ often cited as a good starting value for calibration^38,39^. Alternatively, $\alpha _{\rm{turb-h}}$ can be considered equal to 0 when horizontal fluxes in the water body are deemed not of relevance for the physical process of study or if the vertical diffusion combined with horizontal advection account for horizontal diffusion transport convincingly^39–41^.

For this study, the $\alpha _{\rm{turb-h}}$ value is neglected for the base cases. Given that the model does not consider advective transport in the lake, horizontal heat transport will occur only by material conduction in the base cases. Still, the validity of this assumption will be assessed in a sensibility analysis as part of the study. Boundary conditions will be described in detail in an upcoming section.

The heat produced by the shortwave radiation is described by its divergence over the vertical coordinate in each water column (Eq. 3)^35^. The 1D Beer–Lambert’s law can model the shortwave radiation propagation in the lake column^37,42^:

6 $$\begin{aligned} \frac{\partial I}{\partial z} + \mu _{\rm abs} I = 0 \end{aligned}$$

where $\mu _{\rm abs}$ corresponds to the extinction coefficient of the lake water, equal to ${{0.30}}\, {\rm m}^{{-1}}$ in this study, assuming clean lake water^37^. Equation (6) is solved over the z-coordinate, considering the incident radiation at the surface $I_{\rm{surf}}$ as a boundary condition:

7 $$\begin{aligned} I = I _{\rm surf} (1 - r_{\rm s}) e^{-\mu _{\rm abs}z} \end{aligned}$$

where $r_{\rm s}$ is the albedo coefficient, considered equal to 0.08 for open freshwater^43^, which accounts for incoming radiation reduction due to the surface water reflection. The GHE are included in the model as point sources with a prescribed transient heat load $Q_{\rm pipe}$ in terms of watts per metre (${\mathrm{W\,m}}^{-1}$). To calculate the average temperature of the carrier fluid $T_{\rm{fluid}}$, we use a horizontal GHE thermal resistance model, as developed by^44^:

8 $$\begin{aligned} \frac{T _{\rm g-ext} - T _{\rm fluid}}{R _{\rm p}} = Q _{\rm pipe} \end{aligned}$$

9 $$\begin{aligned} R_p=R _{\rm p-IF}+R _{\rm p-HDPE} \end{aligned}$$

where $T _{\rm g-ext}$ corresponds to the average ground external temperature around the pipe, $R_{\rm{p}}$ is the pipe-fluid thermal resistance, corresponding to the sum of the thermal resistance of the conduction along the pipe $R _{\rm p-HDPE}$ and the resistance of the carrier fluid $R _{\rm p-IF}$, calculated as:

10 $$\begin{aligned} R _{\rm p-HDPE} = \frac{1}{2\pi \lambda _{\rm p}}\cdot ln(r/r\_) \end{aligned}$$

11 $$\begin{aligned} R _{\rm p-IF} = \frac{1}{2\pi r\_ Nu\frac{\lambda _{\rm f}}{d_h}} \end{aligned}$$

where $\lambda _{\rm{p}}$ is the thermal conductivity of the pipe, *r* is the outer radius of the pipe, $r\_$ is the inner radius of the pipe, $\lambda _{\rm{f}}$ is the thermal conductivity of the carrier fluid, $d_h$ is the hydraulic radius of the pipe and *Nu* is the Nusselt number of the carrier fluid, calculated using the Gnielinski equation, as implemented in the COMSOL Multiphysics ’PipeFlow Module’^32^. To determine the external temperature, the ’circavg’ operator is used to calculate an average external temperature in a circle of diameter equal to the pipe, around the point load, to avoid increasing pipe temperature for meshes finer than the pipe diameter, as determined in the 3D Heat Transfer and PipeFlow module coupling^32^.

### Model geometry and properties

**Figure 2:**
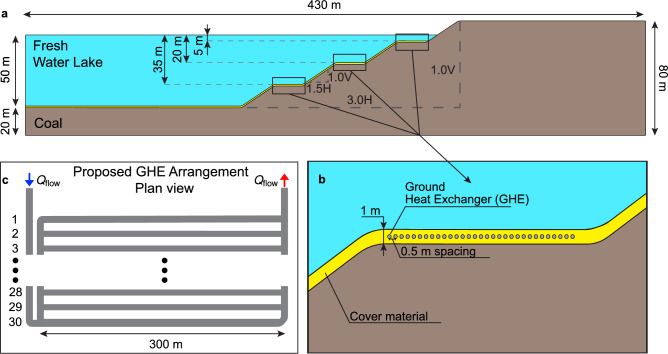
2D pit lake model geometry and material distribution. (**a**) General dimensions of the 2D pit lake model. A Freshwater pit lake rests above a homogeneous coal layer, covered by a 1 m thick cover material to avoid self-combustion. (**b**) A horizontal GHE is laid on the cover material before filling at 0.5 m depth from the pit surface, with 0.5 m of spacing among the collector pipes. (**c**) Plan view of the proposed GHE arrangement, considering a maximum length of 300 m for the assessment, and 30 parallel collector pipes. A blue arrow and a red arrow indicate the fluid inlet and outlet during operation, respectively.

A case study based on the conditions for pit coal mine closure in the Latrobe Valley, located in the Gippsland region, Victoria, Australia, is used to assess the performance of the GHEs in a pit lake environment. The Latrobe Valley subsurface has abundant lignite (brown coal) availability, with lignite to overburden ratios ranging from 4:1 to 5:1^45^. Currently, three lignite mines are set for decommissioning in the upcoming decades. Figure 2a presents the geometry for the pit lake case of study, based on the considered pit wall slope required for closure in the Latrobe Valley mining area^46^. The ground stratigraphy is simplified as a homogeneous layer of brown coal (lignite). Above the coal, a 1 m layer of cover material is considered inside the pit (Fig. 2a, b), which would minimise the risk of coal self-combustion before pit lake filling. The considered cover material geometry is a simplification, whereas, in reality, the cover would only be laid where coal is directly exposed to the atmosphere and where the pit wall slope allows it^46^. At the same time, fill material could be included for other reasons: batter reinforcement, overburden disposal, or as part of slope profiling efforts^47,48^.

During the cover laying process, horizontal GHE would be installed for geothermal energy extraction/injection at 0.5 m from the surface (Fig. 2b) and at three different pit lake depths, 5 m, 20 m and 35 m. The GHE considers an array of 30 horizontal pipes connected at both ends, with a 0.5 m spacing, as seen in Fig. 2c, with an additional header pipe (see left) to create a self-balancing reverse return configuration to allow evenly distributed flow rate among the parallel pipes. The properties of the GHE are summarised in Table 1. Material properties of the model were obtained from literature and are summarised in Table 2.

**Figure 3:**
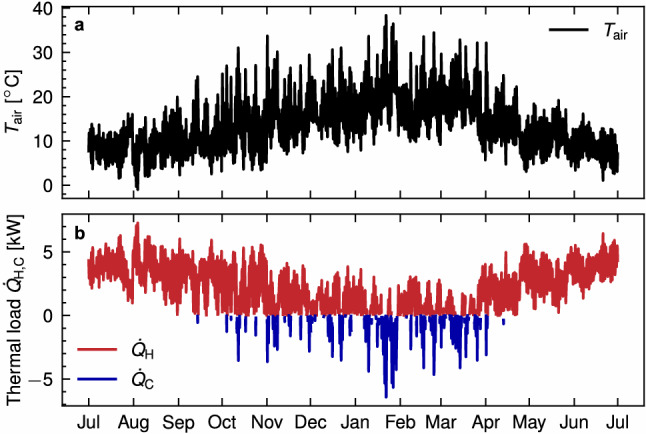
Hourly time series inputs for the assessment. (**a**) Air temperature in the location of interest. (**b**) Representative residential thermal load for the same location, heating demand $\dot{Q}_{\rm{H}}$ in red and cooling demand $\dot{Q}_{\rm{C}}$ in blue.

As input for the model, weather forcing hourly data was obtained from the freely available datasets ERA5 and ERA5-Land^49,50^ for the Latrobe Valley (latitude: 38S to 38.5S, longitude: 146 to 147), and used for the lake thermal dynamics model (see ‘Model validation’ subsection) and the boundary conditions of the model, where appropriate. For the heat exchanger thermal load, a residential thermal load developed for a standard single-family house in the Latrobe Valley is used^20^. Given its residential nature in a cold climate, the thermal load is unbalanced towards heating, with an annual heating demand of 18.7 MWh, compared to only 1.2  MWh of cooling. The air temperature and the thermal load are shown in Fig. 3a, b, respectively.

As hinted by Fig. 3, the system’s operation is considered to begin on the 1st of July (heating season in the southern hemisphere), which matches the ongoing mixing process in the monomictic pit lake, with a close to uniform temperature. Each set of GHEs operates independently, with an average $T_{\rm{fluid}}$ among the pipes, calculated for the GHE at each depth, reflecting the carrier fluid mixing occurring in the GHE before the ascent. The model considers the operation of the system for five years to assess the accumulating effect that the unbalanced thermal load may have on the ground/lake temperatures. The thermal load prescribed to each point heat source is scaled using a load parameter $f_{\rm{load}}$ that is adjusted uniformly for all point sources and for each model, so the system extracts the maximum available thermal energy without exceeding the allowed range for ground and water temperatures (${{0}} \, ^{\circ }{\textrm{C}}$ to ${40} \, ^{\circ }{\textrm{C}}$) during the operation time. From the $T_{\rm{fluid}}$, nominal COP values can be calculated:

12 $$\begin{aligned} COP = \frac{T _{\rm H}}{T _{\rm H}-T _{\rm fluid}} \eta , \qquad COP < 7.0 \end{aligned}$$

where $T _{\rm H}$ is the HP target heating temperature, considered equal to 328.15  K for the assessment (temperatures must be input in Kelvin) and $\eta$ is the HP quality grade factor, considered equal to 0.55 for heating.

### Boundary conditions and cases of analysis

**Figure 4:**
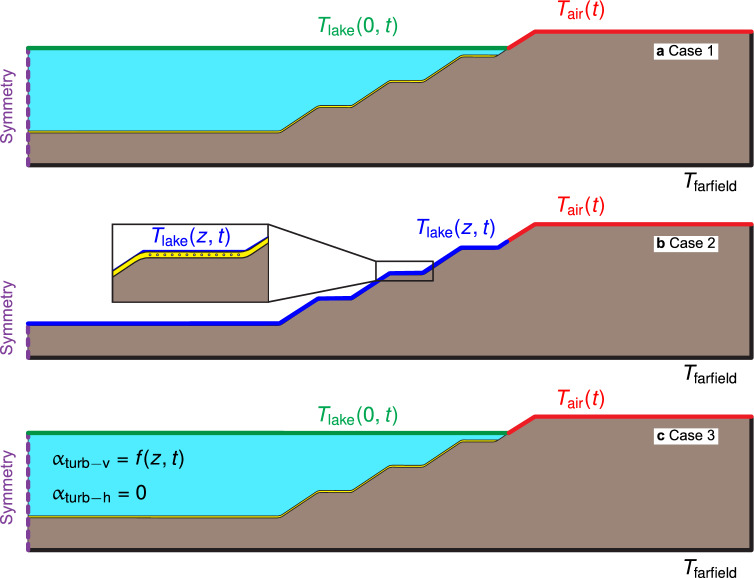
Boundary conditions for each case of analysis. (**a**) Case 1—only material conduction heat transfer throughout the lake. (**b**) Case 2—lake temperature time series as a boundary condition for the ground. (**c**) Case 3—explicit turbulent thermal modelling of the lake, including incident shortwave radiation.

The boundary conditions of each model are presented in Fig. 4. In Case 1 (Fig. 4a), the typical methodology for a buried ground heat exchanger is considered, with a far-field temperature for the ground equal to ${15} \, ^{\circ }{\textrm{C}}$, which corresponds to the representative value reported for the localities of Loy Yang and Traralgon in the Latrobe Valley^51^. An air temperature $T_{\rm{air}}$ time series is used as a boundary condition at the ground surface, a water surface temperature times series obtained from the SIMSTRAT 1D model, as detailed in the calibration subsection, is used for the lake surface, and a symmetry boundary condition is applied at the far side of the lake. In this case, the heat transfer occurs by conduction in the ground and the water, not considering turbulent heat transfer. In Case 2 (Fig. 4b), the lake temperature time series obtained from SIMSTRAT over the whole lake depth is applied as a boundary condition directly onto the ground, so the role of lake temperature stratification is considered, yet not the impact of the GHE operation onto the lake. Finally, Case 3 (Fig. 4c) is like Case 1 but includes the turbulent heat transfer using the vertical diffusivity obtained from the 1D model and the incident radiation onto the water (Eq. 3). A summary of the required inputs to build the models is presented in Supplementary Table S1.

The model utilises a mesh of 34,712 linear triangular elements when the lake is modelled (cases 1 and 3) and 24,319 elements for no explicit model of the lake (case 2) with a minimum skewness factor of 0.5. As a result of the mesh size influence analysis, a minimum element size of 1.0 m is established for the lake domain to ensure a proper mesh resolution to model the steep gradients along the thermocline accurately. Similarly, further mesh refinement of the cover domain around the point sources representing the GHEs is applied to avoid mesh-size effects on the determination of GHE fluid temperatures. Supplementary Fig. S1 includes a visualisation of the utilised mesh. An implicit backward differentiation formula (BDF) method of order two is used for time discretisation^32^, with a maximum time step of one hour. The solution is stored in a 4 h basis for the model overall, with a 1 h resolution for GHE fluid temperature time series. A complete simulation for case 3 (the most expensive) requires 2 h and 46 min of time using 16 CPU Cores (AMD EPYC 7763 64-Core Processor, 2445 Mhz) and 64GB of RAM from a virtual computing machine, and utilises 18.5 GB of storage when solved. As indicated previously, a horizontal diffusivity parameter equal to 0 is considered as a base case. The three cases previously described are the study’s basis, with an additional sensibility analysis where the horizontal turbulent diffusivity $\alpha _{\rm{turb-h}}$ is considered to be $0.001\,{\rm m}^{2}\,{\rm s}^{-1}$ , to assess its relevance in the behaviour of the thermal model and its role.

### Model validation

To confirm that the developed 2D model is appropriate for the study, we assessed its capability to reproduce a representative lake’s thermal dynamics. For this purpose, the 1D finite difference lake model SIMSTRAT^37^ is used. As in the previous section, weather-forcing time series gathered from ’ERA5’ and ’ERA5-Land’ datasets were used as input for SIMSTRAT. The required timeseries for the model include wind speed, air temperature, short and long wave radiation, and vapor pressure (forcing mode 5 in SIMSTRAT). For simplicity, the whole lake is considered to be made of fresh water, with a depth of 50 m. The input pit-lake bathymetry was derived from the study 2D pit geometry (Fig. 3a), using a solid revolution approach to calculate a change of cross-sectional area with depth easily. No water inflows or outflows are considered in the model. The model time step is equal to 360 s and a homogeneous grid of 0.25 m is used for the vertical column. The simulation begins in the southern hemisphere winter (1st of July, 00:00) with homogeneous water temperature conditions equal to ${4}^{\circ }{\textrm{C}}$. A spin-up time of 20 yr is used to let the model stabilise to periodic seasonal temperatures over each year.

**Figure 5:**
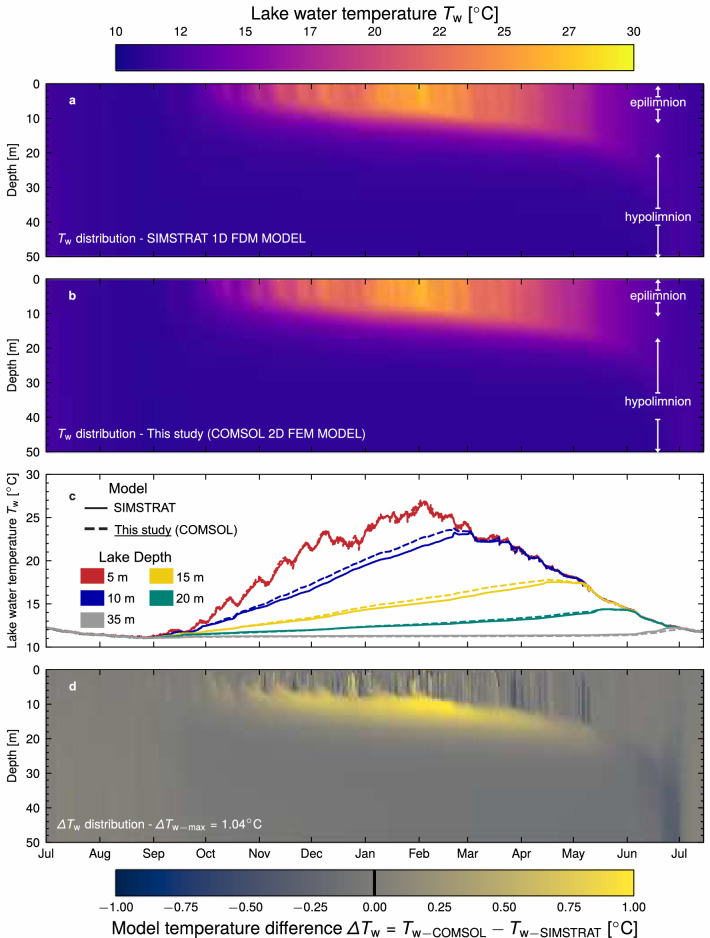
Lake temperature changes over time and depth presented by colour map. (**a**) Results from 1D finite differences (FD) lake model SIMSTRAT. (**b**) Results obtained in this study from the 2D finite element (FE) model in COMSOL Multiphysics. (**c**) Comparison of the water temperature results between both models for 5m, 10m, 15m, 20m, and 35m of depth. (**d**) Temperature distribution differences between both models. The colour map legend for (**a,b**) is shown at the top of the figure while the colour map legend for (**d**) is shown at the bottom. Identified approximate epilimnion and hypolimnion depths are shown on the right side of (**a,b**). A continuous and a dashed line are used in (**c**) for the temperature time series results for the SIMSTRAT and COMSOL (this study) models, respectively. Different colours are used for different depths in (**c**), as shown in the legend on the left side of the panel.

The simulation results in SIMSTRAT after temperature stabilisation are presented in Fig. 5a for one year, where a monomictic lake thermal structure, i.e. the lake undergoes one complete mixing cycle per season, is observed. The lake is thoroughly mixed at the beginning of the simulation in winter (July in the southern hemisphere). Thermal stratification becomes apparent in spring (September), attaining its peak during summer, when water in the epilimnion reaches peak seasonal temperatures, while the water in the hypolimnion remains homogeneously colder. Finally, as the epilimnion cools down, mixing along the lake begins. While monomictic lakes are a possible outcome for filled pit lakes, other thermal structures could occur given variations in the water salinity or its chemical and biological contents, such as meromictic conditions, where deep water layers of the lake do not participate in seasonal mixing^52^, which falls outside the scope of this study.

Moving forward, a $\alpha _{\rm{turb-v}}$ (*z*, *t*) time series, dependant over time and depth, is obtained as an output of the SIMSTRAT model, which is then used in a column COMSOL model as an input to model turbulent thermal transfer. The column model is built as a 2D rectangular (50 m of depth and 1 m of width) domain of freshwater. Turbulent thermal heat transfer and incident solar radiation are included in the model. Surface lake temperature is used as a temperature boundary condition, as in Case 3 (Fig. 4c), while thermal insulation is considered for the lateral and bottom boundaries. A rectangular mapped mesh of maximum element size of 0.25 m is used for the column, with a time step of 1.0 h. Further mesh or time discretisation refinement was found not to have an impact on the results. The effect of the geothermal flux is implemented as in SIMSTRAT^37^, applying a distributed geothermal flux over the depth equal to ${{\mathrm{0.10}}}\,{\mathrm{W \, m}}^{-2}$, that depends on the lake cross-sectional area. The geothermal flux was found to have a negligible impact on the temperature distribution over time and depth, in agreement with the results found in the literature for deep lakes^53^. A latitude equal to −38.15(S) and air pressure of 965 mbar (96.5 kPa) were assigned to the SIMSTRAT model. The same albedo and extinction coefficient values reported in the previous sections were used in the validation. Other required parameters by SIMSTRAT were left as default values.

The results for the column model are shown in Fig. 5b. The results for this study’s COMSOL column model follow the monomictic thermal behaviour of the lake during the season. Further comparison of both models (Fig. 5c) reveals close similarity among both models at the depths of GHE location, which are of interest for the study. The most significant differences occur around 10 m of depth from the lake surface, reaching a maximum around ${1.0}^{\circ }{\textrm{C}}$ in February (summer). Figure 5d further illustrates the differences in simulated lake water temperature $\Delta T _{\rm w}$ between both models over depth and time, simply defined as:

13 $$\begin{aligned} \Delta T _{\rm w} (z,t) = T _{\rm w-COMSOL} - T _{\rm w-SIMSTRAT} \end{aligned}$$

where $T _{\rm w-COMSOL}$ and $T _{\rm w-SIMSTRAT}$ are the water temperatures estimated by COMSOL and SIMSTRAT, respectively. A positive $\Delta T _{\rm w}$ value indicates an overestimation of $T_{\rm{w}}$ by the COMSOL column, while a negative value indicates the contrary. The maximum $\Delta T _{\rm w}$ value is ${1.04} \, ^{\circ }{\textrm{C}}$ obtained at 10.8 m of depth. The maximum discrepancies between both models are mainly found between the epilimnion and the hypolimnion (thermocline) during the summer season. On the other hand, during the mixing season at the beginning of June, there is an underestimation of the temperature at the hypolimnion with a maximum difference value around ${0.33}\, ^{\circ }{\textrm{C}}$, which stabilises to the same value afterwards. Finally, a staggered pattern is observed in the epilimnion during summer, with brief periods of homogeneous difference in the whole layer.

While the magnitude of the differences over depth and time is limited (between 0 and 10% of the simulated temperature value, and typically no more than 3%), these mostly occur in periods and depths of sharp changes of the $\alpha _{\rm{turb-v}}$ parameter, appearing as a limitation of the methodology. Still, the COMSOL model developed in this study is deemed appropriate for its needs. Furthermore, a comparison using Case 2 with the temperature distribution obtained from SIMSTRAT and the COMSOL column verifies the negligible impact of the temperature differences on the GHEs performance, as illustrated in Supplementary Fig.S2. Additionally, it has been verified that the results for the lake temperature distribution in Case 3, at a sufficient distance from the pit wall, match the results obtained for the column model, as shown in Supplementary Fig. S3.

Finally, to confirm that the 2D point source/thermal resistance model is capable of estimating representative carrier fluid temperatures for the GHE, a validation of the model is carried out using experimental results by^54^ and further 3D numerical modelling performed by^34^, as detailed in the Supplementary Note.

## Results and discussion

### Carrier fluid temperature and lake/ground isotherms

**Figure 6:**
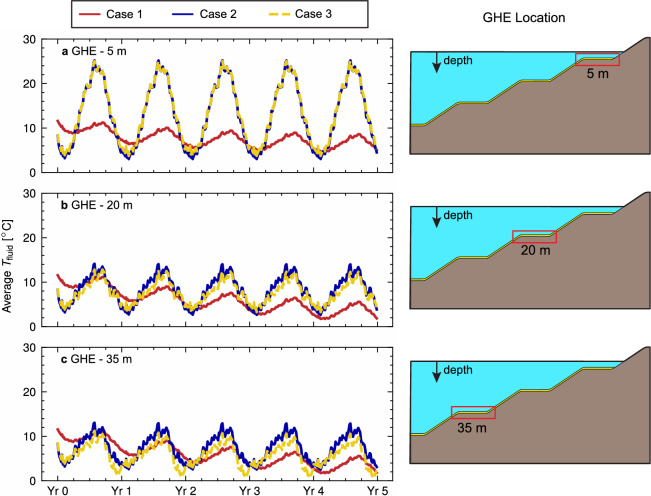
Average carrier fluid over time for GHE at different pit depths, for a 5 year operation of the geothermal system. (**a**) GHE located at 5 m. (**b**) GHE located at 20 m. (**c**) GHE located at 35 m. Hourly time series were resampled to 5 days for clearer visualisation. Time series for cases 1, 2 and 3 are plotted using a red continuous line, blue continuous line and yellow dashed line, respectively. A schematic representation of the GHE is shown to the right of each panel.

Figure 6 presents the results of the numerical model for the base conditions, in terms of the calculated average carrier fluid temperatures $T_{\rm{fluid}}$ in each GHE, for a 5 year operation period. For the shallower GHE (5 m—Fig. 6a), similar results are obtained for cases 2 and 3, while a significant difference with Case 1 is appreciated. A steady downward trend occurs in the $T_{\rm{fluid}}$ for Case 1, while stabilised seasonal temperatures are appreciated for cases 2 and 3. As the depth of the GHE increases (Fig. 6b, c), the $T_{\rm{fluid}}$ downward trend over time (Case 1) increases in magnitude, highlighting a lower availability of thermal energy for heating as depth increases. Given that the heat transfer is assumed to occur only by conduction in Case 1, and the insulation properties of the coal homogeneous layer beneath the GHEs (Table 2), taller water columns separate the deep GHE from the lake surface temperature boundary condition (Fig. 2a). This limits the renewal of the temperature in the ground and lake around the GHE.

For cases 2 and 3, larger seasonal changes in $T_{\rm{fluid}}$ occur relative to Case 1. The amplitude of the $T_{\rm{fluid}}$ variations decreases with increasing GHE depth, emphasising the influence of the stratified water column over the average $T_{\rm{fluid}}$ in the model. Additionally, as the depth of the GHE increases, discrepancies between cases 2 and 3 become more apparent, beginning with very close results for the 5 m GHE (Fig. 6a). In contrast, more significant differences are seen for the 35 m GHE (Fig. 6c). This behaviour is attributed to the reduced thermal diffusivity for the water at the deeper portions of the lake in Case 3, which allows for a cooling effect to occur due to the unbalanced residential thermal load, accumulating along the season. This behaviour is easily observed at the end of each year for the 35 m GHE in Fig. 6c. On the other hand, by considering the lake’s temperature as a boundary condition in the pit surface for Case 2 (Fig. 4b), ’infinite’ low-enthalpy thermal energy is available from the lake. Differences in $T_{\rm{fluid}}$ results are not evidenced in the 5 m GHE, given the high diffusivity found in the epilimnion year-wide. Hence, the lake temperature boundary simplification is a closer match at said depth.

**Figure 7:**
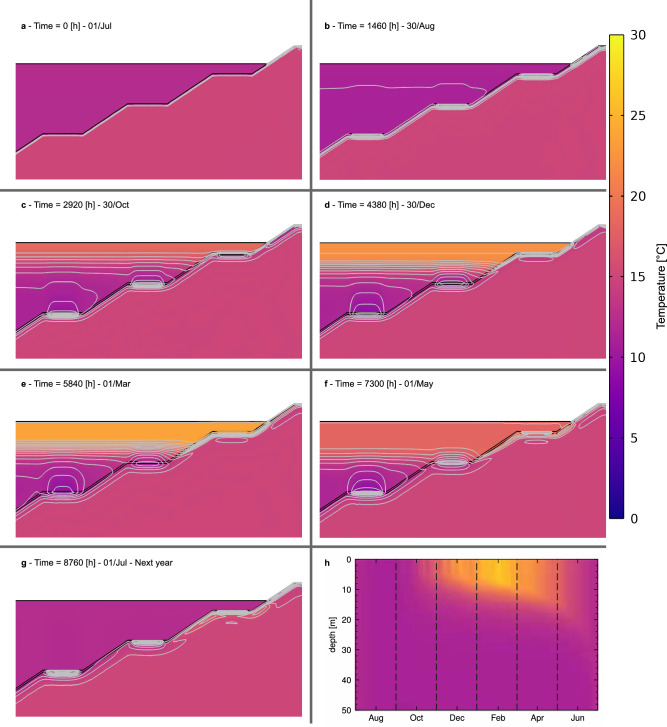
Temperature distribution along the pit lake during the year for Case 3. (**a**) 0 h—01/Jul 00:00. (**b**) 1460 h—30/Aug 20:00. (**c**) 2960 h—30/Oct 16:00. (**d**) 4380 h—30/Dec 12:00. (**e**) 5840 h—01/Mar 08:00. (**f**) 7300 h—01/May 04:00. (**g**) 8760 h—01 July: 00:00, next year. (**h**) Pit lake temperature distribution over the year, each dashed lines signal the selected dates for the plot. Isotherms in (**a–g**) are drawn in continuous grey lines. The colour map legend for all panels is shown to the right of the figure. Caption in hours refers to the time since the start of the simulation (01 July, 00:00).

In contrast with Case 1, the previously highlighted yearly cooling effect for Case 3 (Fig. 6b, c) does not accumulate over the years of operation. The lake mixing process at the beginning of each year allows temperature diffusion along the whole water column into the boundary temperature at the surface. The yearly cooling process is summarised in Fig. 7, for the first year of operation (simulation time range: 0 h to 8760 h). Figure 7a shows the lake and ground with a homogeneous temperature equal to the initial conditions. As the simulation progresses, the lake begins to stratify in spring (Fig. 7c), while the cooling effect of GHE operation at 20 m and 35 m depth becomes apparent. Both phenomena continue to develop in summer (Fig. 7d, e). With the beginning of the overturning effect in spring (Fig. 7f) due to decrease in the temperature in the epilimnion and increased turbulent mixing, the accumulated cooling effect is diffused away for the 20 m depth GHE, with the 35 m GHE following after, as the lake mixes entirely during the winter.

Over the year of operation (Fig. 7a–g), little temperature variation in the coal layer $\Delta T _{\rm coal}$ is observed. This impact is measured through a ’penetration depth’ ($d_{\rm{pen}}$), defined herein as the depth from the GHE where a change of temperature at least ${1.0}\, ^{\circ }{\textrm{C}}$ occurs due to the GHE operational effect. After 5 years, maximum differences of ${8.3}\, ^{\circ }{\textrm{C}}$ relative to the far field temperature and a maximum temperature penetration depth ($d_{\rm{pen}}$) of 10.0 m occur for the 35 m GHE in the coal layer for Case 3. The temperature variation $\Delta T _{\rm coal}$ and penetration depth $d_{\rm{pen}}$ were also measured for cases 1 and 2 for comparison, and all values are summarised in Table 3. As expected, the availability of lake thermal energy is inversely proportional to the energy extracted from the ground. Then, the maximum $\Delta {T} _{\rm coal}$ values are found for the coal layer in Case 1, increasing with GHE depth. Regarding $d_{\rm{pen}}$, the magnitude of differences among different cases is less significant, while similar trends to $\Delta {T} _{\rm coal}$ are observed.

### Thermal efficiency and available energy

**Figure 8:**
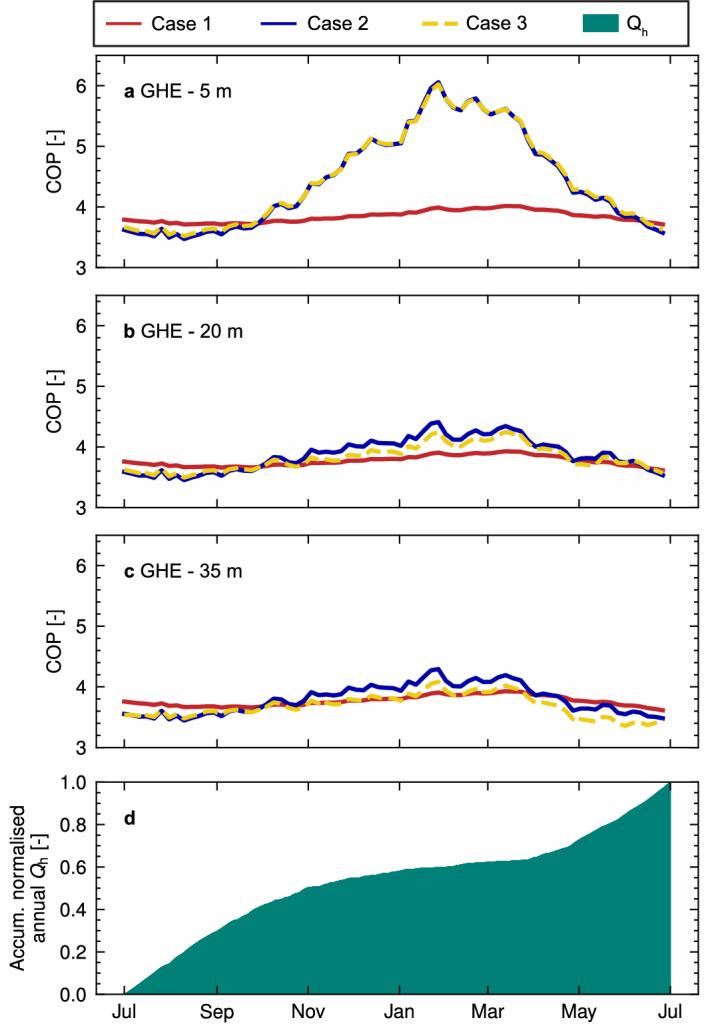
Heating COP over time for GHE at different pit depths. (**a**) GHE located at 5 m. (**b**) GHE located at 20 m. (**c**) GHE located at 35 m. (**d**) Accumulated normalised heating demand per GHE over the year. Hourly time series were resampled to 5 days for clearer visualisation. Year No. 3 of operation is shown since it is the first year when the temperature variations are stabilised for Case 3. Time series for cases 1, 2 and 3 are plotted using a red continuous line, blue continuous line and yellow dashed line, respectively.

The heating nominal COP was calculated based on the obtained average $T_{\rm{fluid}}$ per GHE using Eq. (12), for a complete year of operation, as visualised in Fig. 8. Since the target temperature for heating is kept constant during the year, the COP value depends directly on the obtained $T_{\rm{fluid}}$ and thus shows similar trends to Fig. 7. The most significant heating COP difference occurs for the 5 m GHE (Fig. 8a) among cases 1 and 2–3, due to the increase in the epilimnion temperature in spring and summer, accounted for in cases 2–3. Significantly lower variations in the nominal COP are observed for the deeper GHEs (Fig. 8b, c), hence the previously identified cooling effect has a minor incidence on the estimated heating efficiency of the system when comparing cases 2 and 3.

Figure 8d shows the accumulated normalised heating demand $Q_{\rm{H}}$ (Fig. 8d). The largest COP values match the plateau in heating demand in summer (November to March). It follows that the actual effective seasonal COP of the operation, the COP during hours of heat extraction, does not differ significantly among cases, as summarised in Table 4. Thus, for the specific purpose of residential thermal loads, differences in the estimated thermal efficiency using an effective seasonal COP are not significant (maximum difference of 0.40 between averages). Yet, suppose the energy available in the epilimnion was used during summer for storage or by consumers with a more uniform heating demand, differences between cases 1 and 2–3 increase (maximum difference of 0.7 between averages). In the case of cooling, A nominal COP of 6.0 was obtained for all the results, considering a cooling objective temperature of ${7}\, ^{\circ }{\textrm{C}}$ and a machine scale down factor $\eta$ equal to 0.50, which highlights the efficient cooling potential of lakes in temperate climates, justified as well by their use as a free cooling source^24^.

**Figure 9:**
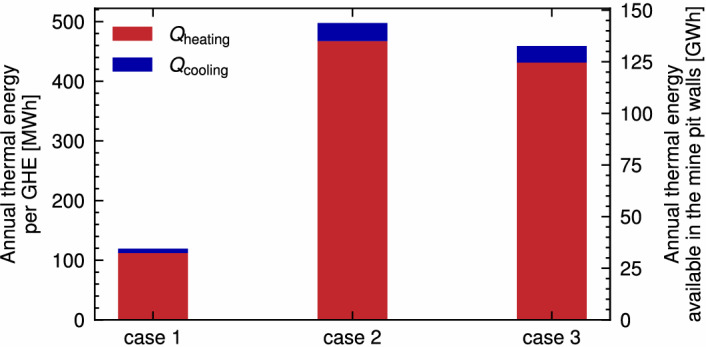
Available annual thermal energy (heating and cooling) per GHE for each case of analysis. Annual heating demand $Q _{\rm heating}$ and cooling demand $Q _{\rm cooling}$ are shown in red and blue, respectively. The right axis indicates the total annual thermal energy potentially available in a representative Gippsland open-pit mine, which could fit about 289 GHEs of 300 m of length. Simplified calculation done considering a pit surface area of 2540 ha, pit depth of 120 m and lake depth of 68 m^46^, considering GHEs of 300 m length).

The annual thermal energy obtained per GHE and for a prospective pit mine in the Latrobe Valley area is presented in Fig.  9. The heating and cooling energy ratio is kept constant for all simulations, scaled proportionally by the factor $f_{\rm{load}}$. For the whole pit lake energy estimation, publicly available geometrical data for the Hazelwood mine in the Latrobe Valley was utilised^46^. An estimated value of 289 GHEs (length equal to 300 m) could be installed in the pit walls. In contrast to thermal efficiency, the selection of modelling methodology greatly impacts the available energy. An annual budget of 117   MWh and 34 GWh per GHE and the whole pit lake is obtained for Case 1. This amounts to 24% and 25% of the budget available using Case 2 and Case 3, respectively.

The trend observed in Fig. 9 is explained by the residential thermal load, which skews significantly towards heating (ratio of heating to cooling of 15.6:1.0), and the low temperatures found in the lake, which limits heat extraction due to the need to avoid freezing temperatures around the GHE and the lake. Given the above, the mechanism for heat diffusion and renewal of the ground and lake temperatures becomes relevant, which explains the poor thermal budget for the case where only conduction heat transfer is considered in the lake (Case 1, only 24% of available thermal energy compared with Case 2). Finally, a minor difference in the available thermal energy among cases 2 and 3 is observed, attributed to the previously described seasonal cooling effect occurring in Case 3.

As mentioned, the magnitude of the variations in the available thermal energy depends on the considered load profile and the pit lake’s year-round temperatures. At the minimum, a larger cooling load could be considered until the thermal load is balanced along the year for the epilimnion, and probably even more as the depth of the GHE increases. On the other hand, for pit lakes located in colder climates, where hypolimnion temperatures vary around ${4}\, ^{\circ }{\textrm{C}}$ to ${5}\, ^{\circ }{\textrm{C}}$, heat extraction would be limited severely if temperatures are bounded above water freezing point.

### Role of lake horizontal heat transfer

**Figure 10:**
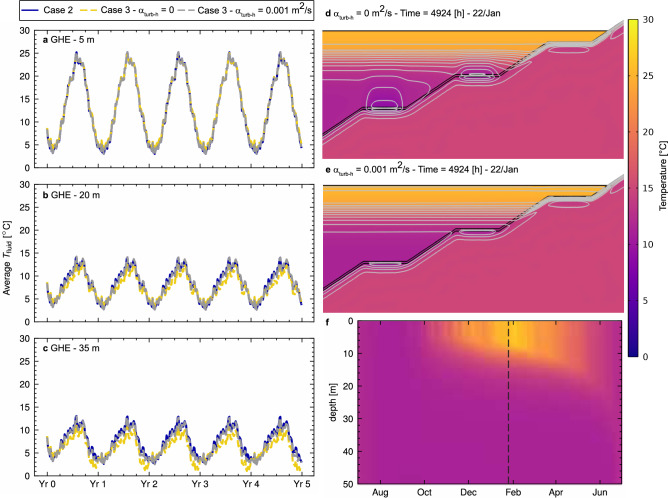
Average carrier fluid temperature $T_{\rm{fluid}}$ over time for GHE at different depths, including horizontal advection sensibility case and temperature distribution colour map for a period of maximum cooling demand (4924 h—22/Jan 04:00). (**a**) GHE located at 5 m. (**b**) GHE located at 20 m. (**c**) GHE located at 35 m. Colour maps: (**d**) Case 3 $\alpha _{\rm{turb-h}}$ = ${0}\,\textrm{m}^{2}\,\textrm{s}^{-1}$. (**e**) Sensibility case $\alpha _{\rm{turb-h}}$ = ${0.001}\,\textrm{m}^{2}\,\textrm{s}^{-1}$. (**f**) Pit lake temperature distribution over the year, with the maximum cooling demand period marked by a dashed line. Hourly time series in (**a**–**c**) were resampled to 5 days for clearer visualisation. Time series for cases 2, 3 and sensibility are plotted using a blue continuous line, yellow dashed line and grey dashed line, respectively. Isotherms in (**d,e**) are drawn in continuous grey lines. The colour map legend for (**d,e**) is shown to the right of the figure.

So far, Case 3 only considers turbulent diffusion for heat transfer in the vertical direction. As a sensibility analysis, an additional run is executed considering explicit modelling of the lake with a horizontal turbulent diffusion parameter $\alpha _{\rm{turb-h}}$ equal to ${{0.001}}{\textrm{m}}^2/{\rm s}$. Figure 10 summarises the results obtained for the average $T_{\rm{fluid}}$, for the GHE at each depth, for cases 2, 3 and the additional sensibility case. As expected, no difference is observed among all cases for the 5 m GHE. As the GHE depth increases, the cooling effect observed for Case 3 is significantly lower in the sensibility case. Moreover, the $T_{\rm{fluid}}$ behaviour in the sensibility case is closer to Case 2, which represents an unlimited supply of thermal energy from the lake, highlighting the ’refreshing’ role of the horizontal diffusion in the thermal budget available for the GHE.

Figure 10d, e present the temperature distribution and isotherms for Case 3 and the sensibility case, respectively. Simulation time is equal to 4924 h, corresponding to the highest cooling demand period in summer, with a strong stratification along the vertical direction of the lake (Fig.  10f). The GHE cooling effect in Case 3 is visualised (Fig. 10d) for the 20 m and 35 m depth, while entirely horizontal isotherms are observed in the sensibility case at all depths (Fig. 10e). For the modelling condition considered in the study, the appropriateness of the simple lake temperature boundary condition (Case 2) is then dependent on the circulation around the embedded GHE area. As a reference, a small value for $\alpha _{\rm{turb-h}}$, equal to ${0.001}\,\textrm{m}^{2}\,\textrm{s}^{-1}$ is enough to attain alike behaviour to Case 2, considering that ${10}\,\textrm{m}^{2}\,\textrm{s}^{-1}$ is the default suggested values for the parameter in geophysical modelling software, as discussed in previous sections.

### Limitations of the study and future works

Several assumptions have been undertaken to facilitate the execution and assessment of the study. The main simplifications for the model are spatial dimensions (2D instead of 3D), model geometry, material properties and their distribution, and one-way coupling in lake turbulent thermal behaviour. A 3D study would allow a more accurate representation of the pit and the potential embedded GHE arrangement at the cost of being more computationally expensive. Similarly, for a more detailed assessment, more information about model geometry and material properties and distribution could provide more accurate results regarding GHE thermal performance, although making it difficult to extrapolate to other cases. Regarding lake thermal stratification modelling, a two-way coupling could provide more detailed data regarding the impact of embedded GHE in changes in the lake’s turbulent thermal stratification. Results show that when horizontal transport is present, there is negligible influence of the geothermal system operation in the lake temperature distribution (Fig. 10d, e), for the conditions in consideration, which agrees overall with literature regarding the role of geothermal influx in deep lakes in temperate climates^53^ and heat input/output for residential use in lakes with open loop systems^55^.

For the pit lake model, the study considers the following assumptions: the pit lake is completely filled with fresh water (no changes in water level), the lake water is clean of optically active substances throughout the year, the thermal seasonal pseudo-steady state is reached before the geothermal system’s operation begins (thermal variations in the lake are repeated each year), and groundwater flow along the pit walls is disregarded. Variations in the results are expected whenever these assumptions are not valid. Thermal lake dynamics depend on lake water level^35^, which this study has found to be relevant in the GHE thermal performance, particularly regarding the available thermal budget. Moreover, the role of heat advection due to groundwater flow has been studied extensively for borehole heat exchangers and thermo-active structures, with a general agreement on its positive effects on heat transfer due to thermal advection^56,57^.

The embedded GHE shows a significant thermal output and reaches steady state operation quickly thanks to the lake thermal seasonal mixing. Operation of the geothermal system may be desired or needed before the pit lake is filled completely. Design and operation of the GHE during filling must consider significant variations in the lake water level. Moreover, high hydraulic gradients along the pit walls are expected due to the ongoing rebound of the surrounding water table. Given how the filling process can take many years to decades, the role of these phenomena warrants further study. Finally, erosion in the pit walls is often expected during the filling period^58^. The accepted erosion levels during the filling period may be below the optimal depth of the GHE regarding its thermal design or leave certain locations as not feasible for GHE embedment. Similarly, more astringent regulations regarding thermal impact on a water body could impact potential locations for the GHE.

The representation of the lake as a Dirichlet boundary condition (Case 2) can be extended for the development of a 3D numerical thermal model, following available numerical modelling methodologies for horizontal GHEs^31,34^. For a one-way coupling between the lake turbulent transport and the thermal model, an extended 1D multi-column approach could be implemented to account for spatial (horizontal) variations in the lake water column properties^59^. To balance accuracy and efficiency, the geophysical modelling community relies on specific numerical tools for full 3D modelling. These tools, such as the use of (non-cartesian) $\sigma$ grids^38,39^ and mode splitting through differentiated time steps^39^, are not commonly available in the multiphysics software often used for GHE numerical modelling. If a fully 3D lake numerical model is needed (or available), coupling with a thermal ground model with heat flux transfer along its surface boundary should be feasible through a programmed interface between the models. Similarly, direct two-way coupling between the lake and a thermal ground model for the basin would be possible.

## Summary and conclusions

The addition of GHEs to the basin of an engineered pit lake has the potential to generate clean and renewable thermal energy for nearby consumers while coexisting with already-developed mine closure solutions. Difficulties from the requirement of GHE embedment before pit lake filling, the temporal scales of the filling process, and the required lake depths for thermal stratification development limit the possibility of pilot field testing to realise the geothermal potential of the technology. Numerical modelling provides an alternative for assessing thermal performance along the required time and spatial scales, given that the relevant physical processes are correctly accounted for. From a numerical perspective, this study provides a first approach to analyse the thermal behaviour of embedded GHEs in a pit lake. Three different modelling approaches of varying complexity derived from methodologies commonly found in the shallow geothermal and geophysical modelling community are considered.

Considering turbulent heat transfer along the lake is vital to correctly estimate the available thermal energy for sustainable long-term GHE operation. The only-conduction model (Case 1) shows a significantly lower annual thermal budget (up to 34 GWh for a whole pit lake, with 117  MWh per GHE comprising 30 HDPE pipes of 300 m each). In contrast, the model accounting for turbulent heat transfer in the lake (Case 3), has over four times the annual thermal budget (133 GWh and 459  MWh for the whole lake and per GHE, respectively) compared to Case 1. On the other hand, the Dirichlet temperature boundary condition (Case 2) results in an additional 8% of annual thermal budget relative to Case 3.

The thermal efficiency of a heat pump operating downstream does not differ significantly (maximum difference of 0.7 COP between cases 1 and 3). At the same time, accounting for the lake as a simple Dirichlet temperature boundary condition (Case 2) shows negligible differences with the more computationally expensive explicit modelling of the thermal stratification of the lake (Case 3), for GHEs closer to the epilimnion. This is explained by the high mixing rate at shallow lake waters and supports the modelling considerations found in the literature for an embedded GHEs in shallow water body basins.

When the turbulent thermal transfer in the lake is accounted for explicitly (Case 3), a cooling effect along the water column (given the unbalanced thermal load) is observed as the depth of the GHE increases. Seasonal lake mixing limits the accumulation of the cooling effect. This behaviour makes the lake a suitable recipient of unbalanced thermal loads compared to common GHE laid in the ground, where no additional sources of thermal energy renewal are found. It must be noted that this study has focused on providing thermal demands for residential consumers, yet significant potential for cooling is expected, as recognised in the literature.

Allowing even small values for horizontal transport (relative to usual values suggested in the literature) in the model limits the cooling effect of the GHE in the lake. These results validate the simplification of the lake as an ’infinite source’ and, correspondingly, using a simple Dirichlet temperature as a boundary condition for assessing thermal loads of similar shape and magnitude, as long as enough circulation is present where the GHEs are laid.

This numerical study has focused on the thermal performance of embedded GHE in a pit lake basin for varying methodologies regarding the representation of the lake in the numerical model. Differences in seasonal heating COP and available thermal budget are insignificant as long as the lake thermal dynamics are accounted for. Thus (Case 2) would be recommended for rule-of-thumb estimation of similar instances when the focus is on the thermal performance of the geothermal system. The authors highlight the following study directions, found in the development of this work, to be the subject of future studies:

- Extension of the methodology to three dimensions to understand variations in performance for the same lake/water body.
- Implementation of two-way coupling between the lake turbulent transport and the GHE system operation, of particular relevance for areas with reduced water circulation.
- Assessment of groundwater flow importance in the thermal performance of the GHEs, of particular relevance for the operation of the geothermal system during pit lake filling.

**Table 1:** Ground heat exchanger model parameters.

Parameter	Unit	Value
Pipe depth	m	0.5
Pipe length	m	300
Volumetric water flow $Q _{\rm flow}$	l/s	26
Pipe SDR	-	11
Pipe external diameter	mm	50
Pipe width	mm	4.55
Pipe internal diameter	mm	40.9
Number of collectors per bench	-	30
Pipe spacing	m	0.5
Pipe thermal conductivity $\lambda _{\rm p}$ ${}^{\rm a}$	${\mathrm{W\,m}}^{{-1}} {\textrm{K}}^{{-1}}$	0.44

${}^{\rm a}$ GHE pipes are modelled considering steady state heat transfer along its radial direction, neglecting transient effects given their small capacitance relative to the surrounding ground and water.

**Table 2:** Finite element model material parameters.

Material	Thermal conductivity $\lambda$	Density $\rho$	Heat capacity $C_p$	Porosity
	${\mathrm{W\,m}}^{{-1}} {\textrm{K}}^{{-1}}$	${\textrm{kg}}\,{\textrm{m}}^{{-3}}$	${\mathrm{J\,kg}}^{{-1}} \textrm{K}^{{-1}}$	-
Coal ${{}^{\rm a,b}}$	0.25	1367	1316	0.45
Cover ${}^{\rm c}$	2.06	2650	840	0.30
Water ${}^{\rm d}$	0.62	994	4179	–

${}^{\rm a}$ Thermal properties of the granular material correspond to the solid portion of the soil. Bulk properties were calculated considering the soil saturated with water, where appropriate.

${}^{\rm b}$ Thermal conductivity and heat capacity parameters obtained from^60^ for lignite (brown coal). Density and porosity values derived from^61^ specifically for lignite found in the Latrobe Valley, VIC, Australia.

${}^{\rm c}$ Cover properties obtained from literature for wet silty sand - sandy silt^62^.

${}^{\rm d}$ Water properties selected to match a material thermal diffusivity $\alpha _{\rm{mat-w}}$ equal to ${1.5E-7}\,\textrm{m}^{2}\,\textrm{s}^{-1}$ as set by default in SIMSTRAT^37^. Values obtained from COOLPROP database^63^, for H_2_0 with temperature *T* = ${36}\,^{\circ }{\textrm{C}}$ and pressure *p* = 101.3 kPa.

**Table 3:** Impact of GHE operation in coal strata: Maximum temperatures and temperature penetration depth.

	Unit	Case	GHE depth		
			5 m	20m	35m
Maximum temperature difference $\Delta T _{\rm coal}$ at 1 m${}^{\rm a}$	${}^{\circ }{\textrm{C}}$	1	8.3	10.3	10.7
		2	4.1	6.2	6.9
		3	3.9	6.6	8.3
Temperature penetration depth $d _{\rm pen}$ ${}^{\rm b}$	m	1	9.3	9.9	10.0
		2	3.1	8.8	9.4
		3	3.0	9.1	10.0

${}^{\rm a}$ Depth measured at 1 m below the GHE location.

${}^{\rm b}$ Defined as the deepest point below the GHE where at least a difference in ${1}^{\circ }{\textrm{C}}$ occurs during the simulation

**Table 4:** Average heating coefficient of performance (COP) per GHE for each case of study.

GHE depth	Nominal COP—case${}^{ \rm a}$			Effective COP—case${}^{\rm b}$		
	1	2	3	1	2	3
m	–	–	–	–	–	–
5	3.8	4.5	4.5	3.8	4.2	4.2
20	3.7	3.9	3.8	3.7	3.8	3.7
35	3.7	3.8	3.7	3.7	3.7	3.6

${}^{\rm a}$ Nominal COP calculated from average $T_{\rm{fluid}}$ per GHE without regard of heat pump (HP) operation.

${}^{\rm b}$ Effective COP calculated from average $T_{\rm{fluid}}$ per GHE considering whether HP is providing heating or not.

### Supplementary Information

Supplementary Information.

## Data Availability

No raw data has been generated for this study. All inputs used for the numerical models have been obtained from the literature and referenced in the manuscript where appropriate. GHE fluid temperature time series obtained from the models has been made available at Figshare https://doi.org/10.26188/25793994. Further simulation output data will be made available on request. The preprocessing of the input data, data analysis, and figure generation was carried out using Python v3.10.10 (https://www.python.org/). Numerical simulations were carried out using COMSOL Multiphysics v6.0.0.453^32^ (https://www.comsol.com) and SIMSTRAT v3.2^37^ (https://www.eawag.ch/en/department/surf/projects/simstrat/).
