# Supplementary material for: The role of pit lake thermal dynamics on the thermal performance of ground heat exchangers
Source: Sci Rep. 2024 Aug 19;14:19191. doi: 10.1038/s41598-024-69225-6 (PMC11333475; doi:10.1038/s41598-024-69225-6)
Supplement: Supplementary file 1 — Supplementary Information. [file 41598_2024_69225_MOESM1_ESM.pdf]

The Role of Pit Lake Thermal Dynamics on the Thermal Performance of Ground Heat Exchangers

Authors: Mauricio Carcamo-Medel, Guillermo Narsilio, Raul Fuentes

Supplementary Tables

<table>
<tr><th rowspan="2">Parameter</th><th rowspan="2">Type</th><th colspan="4">Case of analysis: Range/value</th><th rowspan="2">Source</th></tr>
<tr><th>Case 1</th><th>Case 2</th><th>Case 3</th><th>Case 3 - Sensibility</th></tr>
<tr><td>Air temperature $T_{air}(t)$</td><td rowspan="3">Time series</td><td colspan="4">−1.0 °C to 38.4 °C</td><td>Era5-land[1]</td></tr>
<tr><td>Solar radiation $I_{surf}(t)$</td><td>-</td><td>-</td><td colspan="2">$0\,\mathrm{W\,m^{-2}}$ to $1085\,\mathrm{W\,m^{-2}}$</td><td>Era5-land[1]</td></tr>
<tr><td>Thermal load $Q_{H,C}(t)$[1]</td><td colspan="4">−6.4 kW to 7.3 kW</td><td>Carcamo-Medel et al.[2]</td></tr>
<tr><td>Lake boundary temperature $T_w(z,t)$[2]</td><td rowspan="2">Time series over depth</td><td colspan="4">10.9 °C to 28.4 °C</td><td>SIMSTRAT column</td></tr>
<tr><td>Lake water vert. turb. thermal diff. $\alpha_{turb-v}(z,t)$[3]</td><td>-</td><td>-</td><td colspan="2">$1.5\times10^{-7}\,\mathrm{m^2\,s^{-1}}$ to $2.45\,\mathrm{m^2\,s^{-1}}$</td><td>SIMSTRAT column</td></tr>
<tr><td>Initial temperature - Lake $T_w(z,t=0)$</td><td rowspan="2">Distribution over depth</td><td colspan="4">12.2 °C to 12.3 °C</td><td>SIMSTRAT column</td></tr>
<tr><td>Initial temperature - Cover $T_{g,cov}(t=0)$[4]</td><td colspan="4">12.2 °C to 12.3 °C</td><td>SIMSTRAT column</td></tr>
<tr><td>Farfield temperature</td><td rowspan="4">Constant value</td><td colspan="4">15.0 °C</td><td>Taylor and Mather[3]</td></tr>
<tr><td>Initial temperature - Coal</td><td colspan="4">15.0 °C</td><td>Farfield temperature[3]</td></tr>
<tr><td>Simulation time</td><td colspan="4">5 yr</td><td>Defined in the study</td></tr>
<tr><td>Load ratio f_{load}</td><td>5.1</td><td>1.2</td><td>1.3</td><td>1.2</td><td>Obtained iteratively[5]</td></tr>
</table>

[1] Negative thermal load values account for cooling load.
[2] Temperature boundary is applied over the complete pit wall for Case 2 and only at the lake surface in the other cases.
[3] A minimum value for the turbulent thermal diffusivity equal to the material thermal diffusivity is used in SIMSTRAT as default.
[4] It is assumed that the cover material is in thermal equilibrium with the lake, i.e. the initial temperature is equal to the initial lake temperature at each depth.
[5] f_{load} value is obtained iteratively to extract the maximum heat while limiting ground/lake temperatures in the range of 0 °C to 40 °C.

Supplementary Table S1. Main inputs for the models in each case.

Supplementary Note

The 2D thermal resistance model is validated using experimental data for a horizontal ground heat exchanger (GHE) pilot test carried out at Yildiz Technical University, Istanbul, Turkey, published by Kayaci and Demir[4]. The dataset was also used by Gu et al.[5] to validate their 3D numerical model. Supplementary Fig. S4a shows a plan view of the horizontal GHE, which considered three parallel pipes connected in series, with a length of 40 m each and a spacing of 3 m. Gu et al. validated their model by comparing carrier fluid outlet temperatures T_{out}, as shown in Supplementary Fig. S4c, where a good agreement is appreciated, with maximum temperature differences of 1 °C. The 3D model considered a constant volumetric flow rate and far-field temperature equal to $21.3\,\mathrm{L\,s^{-1}}$ and 13.3 °C, respectively, plus daily average time series for air temperature and carrier fluid inlet temperature T_{in}. Further details for the pilot study and the 3D numerical model may be found in their respective sources.

The geometry used for the 2D model corresponds to a representative middle section (A-A' in Supplementary Fig. S4a) of the 3D numerical model by Gu et al., as detailed in Supplementary Fig. S4b. Each pipe of the horizontal GHE arrangement is modelled as a point source, with a prescribed thermal load time series $Q_{pipe}(t)$ obtained from the results at the middle section of the 3D model. The top side of the domain considers the daily air temperature T_{air} as a boundary condition, while the constant far-field temperature boundary condition encloses the rest of the domain. A good agreement between the 2D and 3D model carrier fluid average temperature T_{avg} is obtained, as shown in Supplementary Fig. S4d. The average fluid temperature obtained in the 2D model is calculated using Equation 8, while calculated in the 3D model as the average of the inlet (T_{in}) and outlet (T_{out}) carrier fluid temperatures:

$$T_{avg} = \frac{T_{in} + T_{out}}{2} \tag{S1}$$

Supplementary Figures

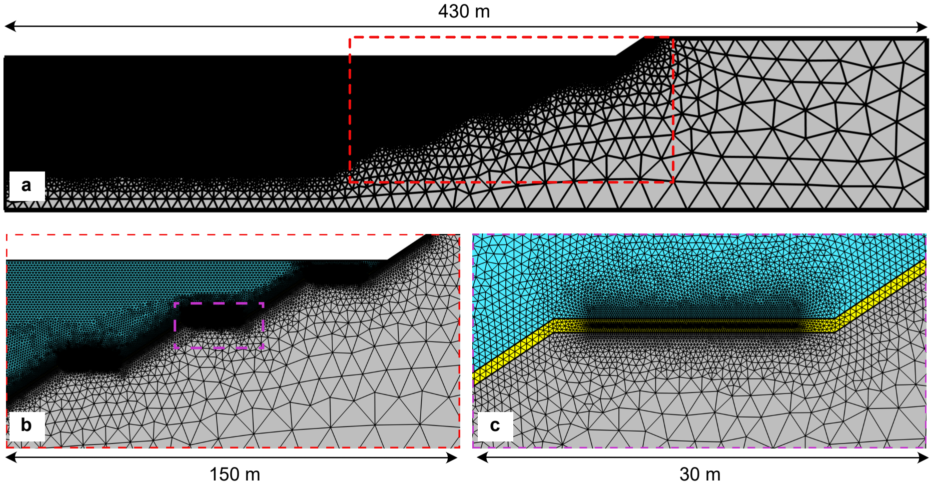

Supplementary Figure S1. Mesh of the model used for the study. (a) Complete model mesh. The close-up shown in panel (b) is highlighted with a red dashed line rectangle. (b) Close-up to the pit wall mesh. The close-up shown in panel (c) is highlighted with a magenta dashed line rectangle. (c) Further zoom in to the mesh around the GHE at 20 m depth.

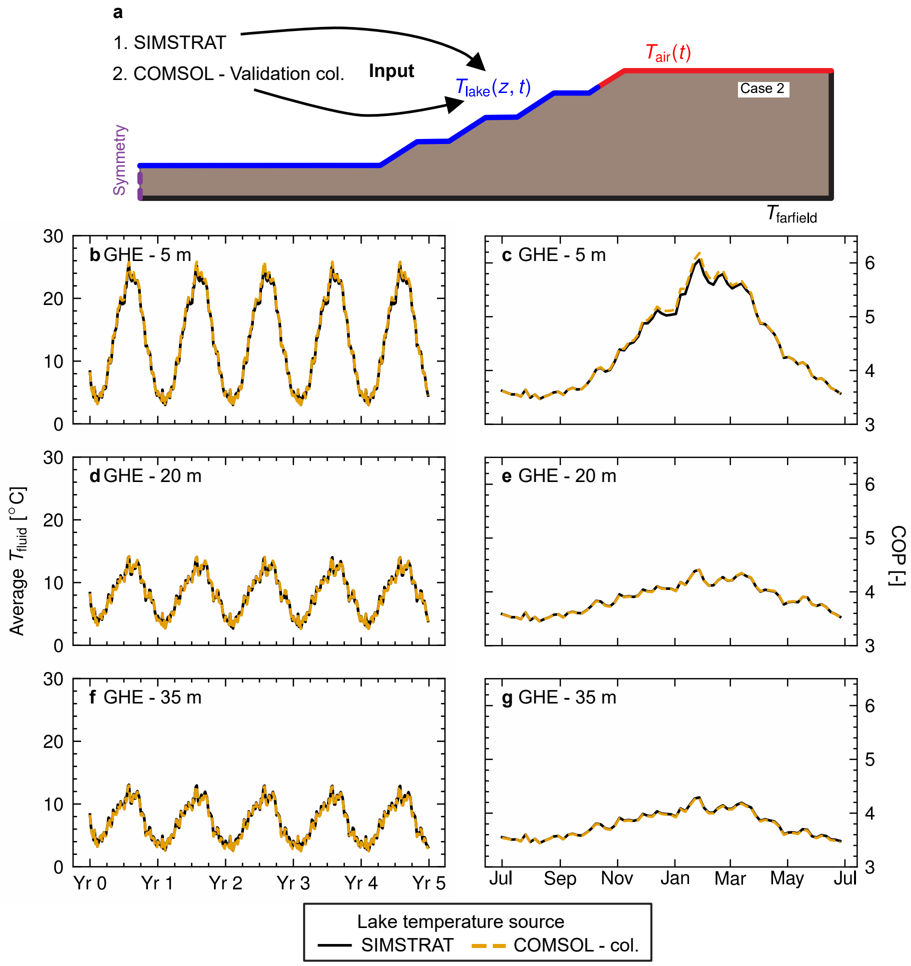

Supplementary Figure S2. Impact of temperature distribution differences on GHE operation. (a) Schematic representation of the input allocation used in a Case 2 type model in the comparison. (b) 5 yr carrier fluid temperature timeseries for GHE located at 5 m. (c) 1 yr COP time series for GHE located at 5 m. (d) 5 yr carrier fluid temperature timeseries for GHE located at 20 m. (e) 1 yr COP time series for GHE located at 20 m (f) 5 yr carrier fluid temperature time series for GHE located at 35 m (g) 1 yr COP time series for GHE located at 35 m. Hourly time series were resampled to 5 d for clearer visualisation. Year No.3 of operation is shown for COP results for consistency with the study. Time series for SIMSTRAT and COMSOL temperature input are plotted using a black continuous line and an orange dashed line, respectively.

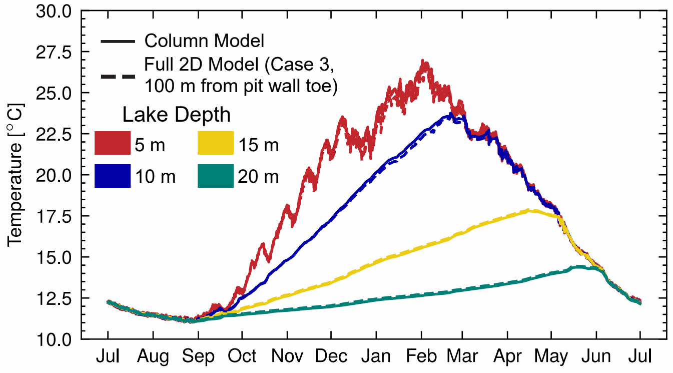

Supplementary Figure S3. Comparison of the water temperature results between both column and complete pit lake models for 5 m, 10 m, 15 m and 20 m of depth. The vertical temperature profile is measured by a vertical cut line in the lake domain, located at 100 m to the left of the toe of the pit wall. A continuous and a dashed line are used for the temperature time series results for the column and complete lake models, respectively. Different colours are used for different depths, as shown in the legend on the left side of the figure.

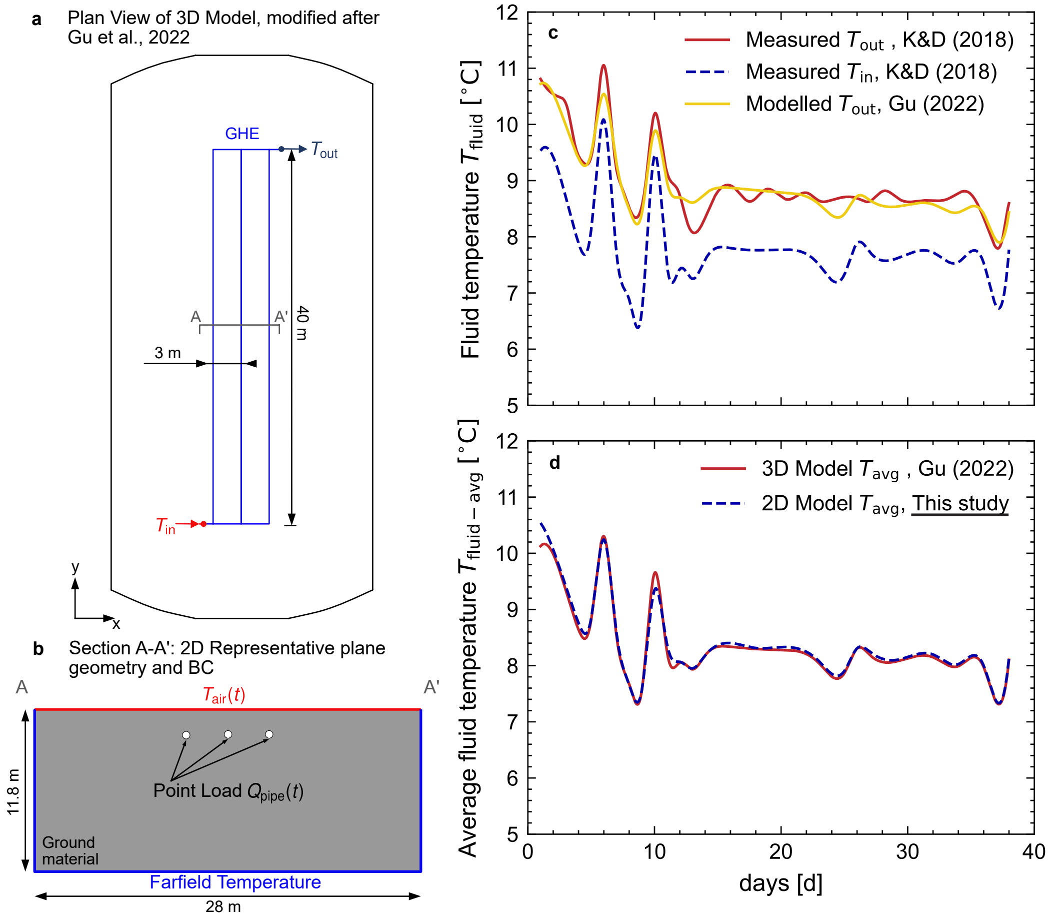

Supplementary Figure S4. 2D resistance model calibration after Kayaci and Demir (K&D, 2018) and Gu et al. (Gu, 2022)[4,5]. (a) Plan view of 3D model used by Gu et al. (b) 2D representative plane and boundary conditions considered for 2D model used for validation in this study. (c) Measured inlet and outlet temperature values by Kayaci and Demir (2018) and outlet temperature values obtained by Gu et al. (2022). (d) Average temperature results obtained by Gu et al. in their 3D model and by the 2D model using the methodology in the present study.

Supplementary References

1. Muñoz-Sabater, J. *et al.* ERA5-Land: A state-of-the-art global reanalysis dataset for land applications. *Earth Syst. Sci. Data* **13**, 4349–4383, DOI: https://doi.org/10.5194/essd-13-4349-2021 (2021).
2. Carcamo-Medel, M., Fuentes, R. & Narsilio, G. Decommissioned open-pit mines are potential geothermal sources of heating or cooling for nearby population centres. *Commun. Earth & Environ.* **5**, 292, DOI: https://doi.org/10.1038/s43247-024-01439-y (2024).
3. Taylor, D. & Mather, D. Geothermal Heat Flow Map of Victoria: Geothermal Atlas Report 1. http://earthresources.efirst.com.au/product.asp?pID=1151&cID=66&c=212746 (2015).
4. Kayaci, N. & Demir, H. Numerical modelling of transient soil temperature distribution for horizontal ground heat exchanger of ground source heat pump. *Geothermics* **73**, 33–47, DOI: https://doi.org/10.1016/j.geothermics.2018.01.009 (2018).
5. Gu, X. *et al.* Geothermal pavements: Field observations, numerical modelling and long-term performance. *Géotechnique* **72**, 832–846, DOI: https://doi.org/10.1680/jgeot.20.P.296 (2022).
